# Understanding Adolescent–Parent Interpersonal Relationships in Youth Sports: A Mixed-Methods Study

**DOI:** 10.3390/sports6020041

**Published:** 2018-05-10

**Authors:** Ausra Lisinskiene, Timothy Guetterman, Saulius Sukys

**Affiliations:** 1 Department of Theory of Sports, Lithuanian University of Educational Sciences, Vilnius 08106, Lithuania; 2 Department of Family Medicine, University of Michigan, Ann Arbor, MI 48109, USA; tguetter@med.umich.edu; 3 Department of Health, Physical and Sports Education, Lithuanian Sports University, Kaunas 44221, Lithuania; saulius.sukys@lsu.lt

**Keywords:** youth sport, parents, adolescents, mixed-methods study

## Abstract

The purpose of this study was to examine the relationship between participation in youth sport and adolescent–parent attachment. A mixed-method explanatory sequential study design was applied. In the first phase, 648 adolescent athletes and non-athletes completed the Inventory of Parent and Peer Attachment–Revised (IPPA–R). In the second phase, 15 adolescent athletes took part in semi-structured interviews. In the first, quantitative phase, three factors were predictors of adolescents’ attachment to parents and peers: trust, communication, and alienation. In the qualitative follow-up, three themes emerged: adolescents’ attachment to the sport; adolescent–parent attachment; adolescents’ thoughts about parents. The analysis of the adolescent–parent interpersonal relationship revealed that athlete adolescents’ relations and attachment to parents compared to non-athlete adolescents are more intensively expressed in all scales: trust, communication and alienation. Interviews with adolescent athletes revealed that parent–adolescent interpersonal relationship and attachment to parents is more important at the early period of sporting life, and becomes less appreciable or unwelcome when children gain sporting experience. The study indicated that the form and degree of parental involvement in children’s sporting activities impacts the effectiveness of parent–athlete interpersonal relationships. The degree and the form of parental involvement in children’s sports chosen by the parents are not always appropriate and encouraging, and they are not always supportive of adolescents’ opinions.

## 1. Introduction

Empirical research indicates that parents have the biggest impact on adolescents’ sport participation [1,2,3,4,5] and parents are highly involved in their children’s decision making about sports activities [6]. Researchers have found different roles of parents in children’s sporting activities: Parents become secondary teachers, counselors, managers, referees, financial sponsors, and are important members of the fan group in competitions [7]. Seeking to involve children in sporting activities at an early age, and to fully disclose their potential, it is vitally important to create a favorable environment during the first year of the child’s personality development and parents play a crucial role in this process [2,6,8,9]. By initially providing children the joy of small achievements, eventually, sport becomes a motivating factor for them, an interesting and meaningful activity which later develops into a lifestyle [10] that is passed on to their children’s children, i.e., to future generations. A child’s achievements in sports can be considered as a merit of their parents too [11]. It was found that parents help their children develop skills to cope with the demands of youth sport, and if their children are talented, parents play an important role in helping their children to achieve the highest elite level of sport [12]. However, children who perceive excessive parental expectations have reported heightened pre-competition anxiety, lower self-esteem, and demotivation [13].

Parent and adolescent interpersonal relationships in sport strongly influence adolescent sport involvement, the level of enjoyment and the athletic achievements [3,14]. Parent involvement in youth sport which directly characterizes the parent’s behavior can positively or negatively impact their children’s sporting experiences [2,14]. Parental involvement and behaviour perceived by adolescents depend on early attachment relationships in the family [10,14]. Secure attachment can be protective and provide a foundation for exploration and normal development, while compromised attachment can lead to negative developmental outcomes in these domains [15]. Secure attachment relationships are associated with appropriate social development and the ability to interact with others throughout life, and individuals with insecure attachment are more likely to lack social attitudes [15]. Specifically, children with anxious-avoidant and anxious-resistant attachment styles have been reported to have behavior problems, emotional difficulties, and social incompetence [16,17]. A secure attachment system serves as a foundation for the expression of emotions and communication in future relationships, provides opportunities for self-regulation of affection, and creates potential for resilience [15]. The parent–adolescent interpersonal relationships in this study are analysed based on parent–child attachment importance. Parent–adolescent interpersonal relationships in youth sport depend on the parent–child early attachment in the family, which in turn affect the level and degree of parental involvement in youth sport [14].

Our study is grounded in attachment theory [16]. The theory states that early experiences with primary caregivers (typically parents) influence a child’s future development of close relationships. A central tenet of attachment theory is the notion that early childhood lays the foundations for the development of personality throughout life [10]. Attachment theory views human behaviour regarding a few key motivational or behavioural systems, such as the neurological systems underlying a person seeking and relying on relationships with others, interest and courage to explore the world, and inclination to empathize with and take care of others [16].

Recognizing the psychological importance of children’s relationships with initial caregivers, it was hypothesized by [16] that the internal working models children construct as a consequence of initial attachment relationships will serve to gather future patterns of cognition, affect, and behaviour. Attachment literature [10,15,16,17,18] gives particular importance to childhood relationships with parents in regulating later states of mind about attachment and relationship formation. Specifically, for adolescents those early experiences that enable them to develop a secure attachment model are more likely to develop internal working models for themselves and others that enable them to maintain a positive and healthy relationships with friends; adolescents develop a style of interaction with others that closely reflects the attachment relationship that they experience with caregivers; and finally, adolescents can internalise complex patterns of emotional regulation developed in early attachment relationships and reproduce these strategies in their relationships with their friends [16]. The contention that a secure attachment relationship involves an attachment figure that is perceived to be available and responsive [16] highlights the fact that adolescents’ attachment relationships with key caregivers are likely to reflect the nature of internal working models that may well function as a psychological template during the construction of new close relationships in sport [16]. Results provided evidence that the nature of the adolescent–parent attachment relationship is significantly related to sporting friendship experiences. More secure adolescent–parent attachment characteristics corresponded to more positive sporting friendships. Adolescents construct mental models related to their thoughts, memories, beliefs, emotional and behavioural background about self and others.

The time spent with peers enables them to develop and maintain social relationships outside the family, to be independent of their parents, to build their future adult identity, express their needs, and develop their culture in a group of peers [19]. Therefore, attachment and relationship with parents change in the period of adolescence; young people are becoming more independent from their parents. Researchers have noted that secure attachment to family gives adolescents a more secure emotional basis which they can always rely on [10,14]. Although the interest in researching parental involvement in sporting activities emerged rather recently, scientists have long been concerned about the influence of parents on children’s sports experiences [2,8,9]. The foundation of theory by [3] is that optimal parental involvement is achieved when parents strive to both understand and enhance their child’s experience, recognising that each child is an individual with specific requirements, and the youth sport experience often occurs over an extensive period. To enhance positive interaction between the parents and the child, parents and players need to communicate, trust, and share common goals [14]. Therefore, greater attention is still focused on relatively specific aspects of parental behaviour in children’s sporting activities (e.g., the parents’ role during and after the competition, among others). There is a lack of research covering a broader aspect of adolescents’ relationships with parents [20], which could contribute to revealing the general aspect of parent–child attachment in sports. It is important to find out how and why participation in sporting activities changes the child’s way of thinking and behaviour. No less important is to understand the role of parents in this process, how the role is performed, and how a sporting activity changes not only the children’s but also the parents’ values, behaviour, and communication within the family.

The scientific literature analysis revealed the following aspects of the problem: only a small number of children can continue to participate in sports without moral and financial support from their families. Furthermore, not all parents are interested in sport, and not all of them understand the significance of children’s education in sport [8,9]. Little is known about how parents are influenced by their children’s participation in sport, including motivation and experience [2]. There is a lack of data examining the relation between children’s involvement in sport and their degree of communication in the family. Finally, there is a need to find out how parental participation in the adolescents’ sport can be strengthened and supported, while acknowledging the perspective of the adolescent athlete.

The research questions in this study are formulated from several perspectives. From the quantitative perspective, researchers seek to analyse the links between participation in sporting activities and attachment to parents and peers among athlete and non-athlete adolescents. From the qualitative perspective, researchers seek to analyse how adolescent athletes understand the adolescent–parent attachment and interpersonal relationship in youth sport? Therefore, we had two aims in this study. First, from the quantitative perspective we examined the links between adolescents’ participation in sporting activities and their parent and peer attachment, and hypothesised that adolescent participants in competitive sport would have strong parental attachment. Second, from the qualitative perspective, we aimed to explore the adolescent athlete’s experiences, related adolescent athlete attachment, and interpersonal relationships in youth sport. We used a mixed-methods explanatory sequential design, which begins with a quantitative phase of data collection and analysis, followed by a connected qualitative phase of data collection and analysis, in order to gain a better understanding of a complex relationship between the adolescent and the parent. The mixed-methods study design helped the quantitative results namely trust, communication, and alienation, to be explained in more depth. The main assumption of the mixed-methods research is that qualitative and quantitative research methods ensure a deeper analysis of a research problem compared to a single-method approach [18]. The integration of both forms of research is central to mixed-methods research.

## 2. Materials and Methods

### 2.1. Study Design

**Figure 1:**
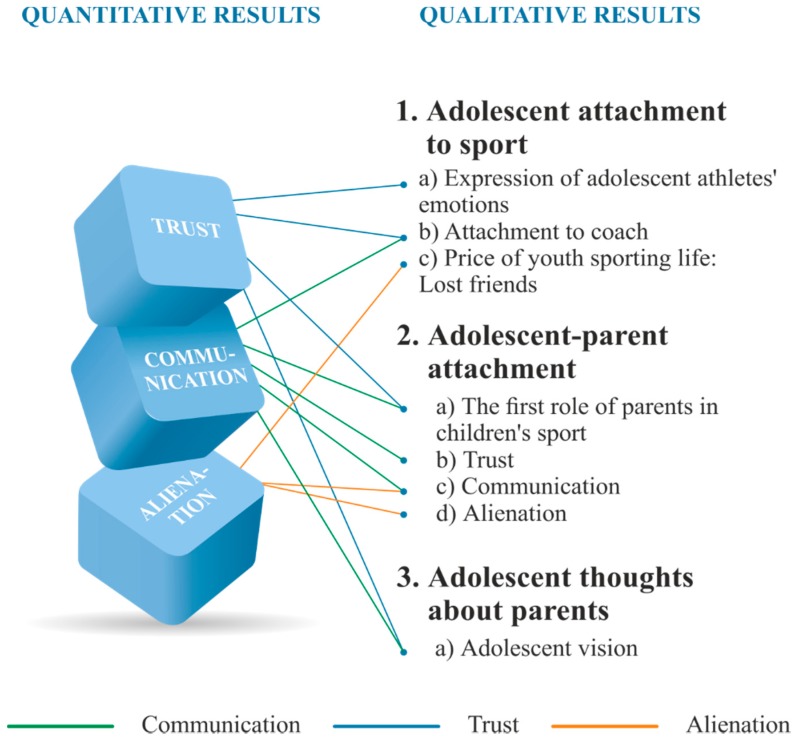
Mixed-methods study: integration of quantitative and qualitative research.

We used a mixed-methods approach [21] to collect, analyse, and integrate both quantitative and qualitative data at some stage of the research process within a single study [21,22,23]. The main assumption of the mixed-methods research is that quantitative and qualitative research methods ensure a deeper analysis of a research problem compared to a single-method approach. In this way, the value of the different approaches to research (e.g., trends as well as the stories and personal experiences) can contribute more to understanding a research problem than one form of data collection (quantitative or qualitative) could on its own, especially when analysing the complex adolescent–parent interpersonal relationship. An explanatory sequential mixed-methods approach was utilized for this study [21]. In this design, the quantitative numeric data are collected and analysed first, followed by the collection and analysis of the qualitative data, which helps explain or elaborate on the quantitative results obtained in the first phase. Then, a qualitative phenomenological interpretative analysis approach (IPA) [24], was used to explain why certain factors, namely trust, communication and alienation tested in the first phase, were significant predictors of the relationship between participation in youth sport and adolescent–parent attachment. The choice to conduct IPA was reasonable for conducting rich and reflective adolescent personal experiences, and for explaining quantitative results. Other common focuses for IPA are the trends reflecting both phenomenological and interpretative aspects. Researchers analyse and interpret the lived experience of the research participant. In this sense, researchers engaged in a double hermeneutic because the researcher is trying to make sense of the participant, and trying to make sense of what is happening to them. This captures the dual role of the researcher. Thus, the quantitative results provided a general picture of the research problem, while the qualitative data and its analysis refined and explained those statistical results by exploring the participants’ views in greater depth. The results of the quantitative and qualitative stages were integrated into the discussion of the outcomes of the entire study (see Figure 1).

### 2.2. Quantitative Phase

#### 2.2.1. Participants and Procedures

Data for the quantitative study were obtained from students at seven schools in the eastern part of Lithuania. A multi-stage sampling technique was used. During Phase I, we selected schools from all schools in the region. In Phase II, we selected classes from those schools by simple random sampling. In Phase III, we invited all schoolchildren from each selected class to participate in the survey. The research sample included 648 adolescents (346 girls and 302 boys) between 15 and 16 years of age (M = 15.6, SD = 0.49). In total, 32.3% (n = 209) of all participants were involved in a competitive sport and had been playing sports for an average of 8.25 years (SD = 2.87). In total 63% of athletes were involved in a team sport, and 37% of athletes were involved in individual sport.

#### 2.2.2. Measures

For the first quantitative phase, a cross-sectional survey design was used. The survey instrument was the Inventory of Parent and Peer Attachment-Revised (IPPA–R) [25]. The IPPA was developed by Armsden, Greenberg (1987) [26] and consists of 25 items across three dimensions: mother, father, and peer As it was designed for use with older adolescents, we employed the shorter version (IPPA–R), which was revised for use with children and early adolescents and does not distinguish between mother’s and father’s attachment. The instrument consists of 28 items measuring parent attachment and 25 items measuring peer attachment, with responses made on a 3-point Likert scale, ranging from 1 (never true) to 3 (always true). The items are grouped into three subscales: trust, communication, and alienation. Responses to negatively worded items are reverse-coded before calculation. The total score for each parent and peer attachment scale is calculated by adding the trust and communication subscale scores, and then subtracting the alienation subscale score [25,26]. The instrument has adequate internal consistency, with Cronbach’s alpha coefficients ranging from 0.74 to 0.91 for the parent attachment items, and from 0.69 to 0.89 for the peer attachment items. Students’ participation in sport was assessed using the question ‘Do you participate in competitive sport?’ With two multiple-choice answers: ‘Yes I am actively involved in sport, I go to sport classes at least three hours a week, participate in competitions and these activities have been lasting for at least two years’; ‘I exercise only in PE classes and in my free time. However I do not go to regular sport classes and do not seek the maximum sport results’. This question has evidence of validity with adolescents through earlier studies [14,27]. Based on their responses, participants were divided for data analysis into two groups: athletes (those who took part in sport at schools or clubs for no less than 3 h per week, and had been participating in the competition for no less than four years) and non-athletes. We specifically identified athletes in this study as athletes who had been involved in competitive sport for no less than four years to gain a deeper understanding of athletes with higher sports experience. This question was validated in the study of [14].

#### 2.2.3. Data Analysis

To conduct the data analysis, we used SPSS version 22.0 (IBM, Chicago, IL, USA). To calculate Cronbach’s alpha coefficients, descriptive statistics, independent samples *t*-tests, Cohen’s d effect sizes, Pearson correlation coefficients, and multiple regression coefficients. Statistical significance was set at *p* < 0.05 for all tests. To determine the meaningfulness of differences, we examined partial eta squared and used Cohen‘s multivariate and univariate guidelines of 0.01 (small), 0.06 (moderate), and 0.14 (large).

### 2.3. Qualitative Phase

#### 2.3.1. Qualitative Research Design

Interpretative phenomenological analysis [24] focuses on the lived experience of participants by incorporating phenomenology and interpretation. It shares the aims of idiographic phenomenology, which provides a detailed analysis of elements of the reflective personal and subjective view of individual experiences. IPA moves one step beyond phenomenology (participants’ accounts) and attempts to report on the participant’s experience by considering the researcher’s view of the world during interpretation.

#### 2.3.2. Participants and Procedures

The sample consisted of 15 adolescents who were 16 years of age, participated in sport, and who responded to the advertisement for volunteers. The demographic data of research participants is presented in Table 1. Demographic characteristics included: a number of research participants, adolescent gender, age, race. Moreover, the demographic data included a sport type: individual/team sport. Finally, we included sport experience, which means adolescent involvement in competitive sport and a involvement in years. Competitive sport, as mentioned in the quantitative methods section, means taking part in sport classes no less than three times per week, participating in competitions, and seeking a sport’s result. Participants were involved in sports at the time of the interview and had eight years of involvement in sports and competition experience.

#### 2.3.3. Interview Protocol Development

The questions given to the research participants focused on the issues raised by the investigation. The protocol served only as a guide in each interview to prevent the researcher from uncontrolled deviation from the analysed topic, and to restrict free associations of the participants and the content of the narrative. Semi-structured interview questions began after the lead researcher established consent. Next, the participants shared more about themselves, their families, and hobbies. Later, more sensitive questions related to the research emerged. The interviews concluded with a neutralizing inquiry about their feelings after the meeting, and an opportunity to ask questions to the researcher.

#### 2.3.4. Data collection

Fifteen stories of adolescent athletes were voice recorded. Research participants were selected based on the following selection criteria: homogeneity, information coverage, and informed consent to participate in the survey. With the help of administrators of sport clubs, information about the planned survey was announced in 7 sport clubs in Kaunas, Lithuania. Only those interested in the survey (parents and athletic children) collected flyers containing the description of the survey and researcher’s contact details. Regarding the sensitive topic and participants’ age, researchers carefully planned the research process in the following stages. First, researchers obtained ethical approval from Lithuanian University of Educational Sciences about eligibility to conduct the research. Next, researchers organised a meeting with each parent of an adolescent and provided a detailed explanation about the ongoing research (the ability to accept the invitation and ability to refuse the participation at any time of the research, the importance of accompanying the child to the researcher). The parents and adolescents were given all the researcher’s contacts enabling them to ask questions at any time. Finally, parent consent for adolescents to participate in the study was obtained prior to the planned research. Fifteen adolescent athletes expressed the wish to participate. All 15 respondents were invited for an interview. Interviews were conducted in the first author’s research office, and the schedule was organised in separate individual meetings with athletes, scheduled in advance at a time convenient for all parties. Interviews lasted from 50 min to 1-h. Questions given to the respondents were formulated in accordance with the research problem. The plan of questions was followed in order not to digress from the research issue, and at the same time not to restrict free associations of the respondents and the content of their stories. The protocol served only as a guide in each interview to prevent the researcher from uncontrolled deviation from the analysed topic and to also restrict free associations of the participants and the content of the narrative. Semi-structured in-depth interview questions were asked after brief informed consent was established and acknowledged by the researcher to allow the participants to feel comfortable and settle down. Next, the participants were asked to share more about themselves, their families and hobbies. Later, more sensitive questions were asked related to the research subject, for example, ‘What does sport means to you?’; ‘How important is parental involvement for you?’ The interviews concluded with a neutralising inquiry about their feelings after the meeting and an opportunity to ask questions to the researcher.

#### 2.3.5. Data Analysis

Data analysis was carried out in compliance with the methodological requirements of interpretative phenomenological analysis [24]. The analysis contained the following stages: transcription, analysis, and credibility checks. Each interview was audiotaped and transcribed verbatim [23]. At this stage, the focus was on how the participants talked about themselves: their tone, rhythm, pauses, or changes in topics. IPA requires detailed and comprehensive interview transcription material (text), which is the object of the analysis. Therefore, some essential aspects of participant interactions were noted (laughing, crying, silence, change in mood, etc.). Material was collected in 15 interviews—voice records (more than 17 h) were transcribed into text. It took, on average, one week to transcribe and analyse one interview: 15 workweeks—on average three months—were spent transcribing the oral speech into written text (268 pages of text to analyse). While analysing the results, the coding system of the qualitative study included the changed research participant name, city, and exact sports field. The name of the research participant was changed and identified for example “A1” (where A—the name of participant, 1—the number of interview). Sports field was identified only for individual or team sports without any explanation of what type of individual or team sport the participant was involved in. We followed qualitative research ethical requirements [23,24] in order to ensure and maximize the security of research participants’ identification.

#### 2.3.6. Ethical Aspects of the Study

Research participants participated voluntarily and for no remuneration. They did not receive any misleading information regarding research goals or the form of results presentation. Research was conducted in accordance with the following principles [28]: right to protection from damage; right to safety; usefulness of the study; privacy; confidentiality; and fairness. The ethical principles included obtaining individuals’ consent. The consent included information about the ongoing research. The aim and the purpose of the study was explained. The use of research participant-given information for research purposes was explained. The consent included information about the research participants’ changed names, city, and exact sports field. The right to refuse to participate in the research at any time was explained. In this sense, the research participant would be excluded from the research, and the recorded interview automatically deleted.

Research quality assurance. Researchers followed [29] four principles of research quality assessment: (a) sensitivity to context, (b) commitment and rigour, (c) transparency and coherence, and (d) impact and importance. Applying sensitivity to context, researchers treated their research participants as true research experts, without researchers’ power-play interruption. The interpretation of the results were made by giving participants a voice in the project and allowing the reader to check the interpretations being made. Regarding commitment and rigour, researchers showed a high degree of attentiveness to the participant during data collection and the care with which the analysis of each case is carried out. To conduct an in-depth IPA interview requires a considerable personal commitment and investment by the researcher in ensuring the participant is comfortable, and in attending closely to what the participant is saying. Rigour refers to the thoroughness of the study, for example the quality of the interview and the completeness of the analysis undertaken. The quality of the interviews were planned carefully. The methodological consultations as well as seminars and workshops, the supervision, and training with experts in IPA were attended prior to the research. Each researcher carefully reviewed every interview and critically reflected on each. Applying the principle of transparency and coherence, researchers clearly and carefully described the research process, including how participants were selected, how the interview schedule was constructed, how the interviews were conducted, and what steps were used in analysis. The coherence is seen through themes brought together logically through drafting and redrafting. Finally, impact and importance are broad principles seen through real validity, which lies in whether the findings tell the reader something interesting, important, or useful [29]. Researchers presented a true story of adolescent athletes in relation to their attachment and interpersonal relationships to parents. This current study tells the reader about possible preventions or interventions through parenting in youth sport. Finally, this study tried to understand and share young adolescent athletes’ feelings, hopes, and desires regarding parental involvement in youth sport.

## 3. Results

### 3.1. Quantitative Phase

Descriptive statistics and correlations for the study variables are presented in Table 2. Regarding parent attachment, adolescents reported the highest scores on trust, followed by alienation, and communication. Evaluating the attachment to peers, adolescents scored higher on the trust subscale, lower on communication, and the lowest on alienation. The results revealed that trust, communication, and alienation were positively correlated to each other in parent attachment (Table 2). Significant correlations were also found between all subscales in peer attachment. Adolescents’ involvement in sport was positively correlated with trust in parents and peers, and also with alienation from peers. Sport experience was not correlated with any of the attachment variables. We calculated the differences between adolescent participation in sport and their attachment to parents and peers (Table 3). The results showed that the mean scores of adolescents participating in sport were significantly higher regarding trust in parents subscales compared to non-athletes. Analysis revealed that there were no significant differences when comparing communication and alienation subscales. The study found that athletes scored significantly higher on trust in peers, and alienation with peers subscales. To investigate the effects of participation in sport on attachment to parents and peers, we carried out a regression analyses with gender as a control variable. Because experience with sport was not significantly related to the dependent variables (attachment subscales), we did not control for them in the regression analyses. In the second step, involvement in sport was entered. We conducted four regression analyses with adolescents’ attachment to parents and subsequently with attachment to peers. The first regression was conducted with trust in parents as the dependent variable. This analysis revealed that gender (β = −0.10, t = 2.51, *p* < 0.05) and participation in sport (β = 0.7, t = 1.98, *p* < 0.05) had significant effects on adolescents’ trust in parents (F = 6.45, *p* < 0.01, adjusted R^2^ = 0.02). The results indicate that those who were male, and also participating in sport, had more trust in parents. The second regression analysis was conducted with communication as the dependent variable. The results did not reveal any relationship between gender, participation in sport, and adolescents’ communication with parents. The third regression analysis was conducted with alienation as the dependent variable. The results revealed that gender (β = 0.12, t = 2.88, *p* < 0.01) had a significant effect on adolescents’ alienation from parents (F = 5.41, *p* < 0.01, adjusted R^2^ = 0.02) indicating that males were more alienated from parents. There was no relationship between adolescent’s participation in sport and alienation from parents. The fourth regression analysis was conducted with overall parent attachment as the dependent variable. The results did not reveal any relationship between participation in sport and adolescents’ attachment to parents. Next, we examined the relationship between adolescents’ involvement in sport and attachment to peers. The results indicated that gender (β = −0.19, t = −4.93, *p* < 0.01), and participation in sport (β= 0.12, t = 2.99, *p* < 0.01), had a significant effect on adolescents’ trust in peers (F = 14.97, *p* < 0.001, adjusted R^2^ = 0.04). The research also revealed that gender (β = 0.35, t = 9.42, *p* < 0.001), and participation in sport (β = 0.10, t = 2.65, *p* < 0.01), (F = 45.43, *p* < 0.001, adjusted R^2^ = 0.13) significantly predict adolescents’ communication with peers. The results indicate that those who were female, and also participated in sport, had more trust in peers and communication with peers. Gender (β = 0.13, t = 3.26, *p* < 0.001) and involvement in sport (β = 0.11, t = 2.87, *p* < 0.01) were also significant predictors of alienation from peers (F = 8.36, *p* < 0.001, adjusted R^2^ = 0.03). Lastly, there were significant predictions regarding gender (β = −0.29, t = −7.49, *p* < 0.001) and participation in sport (β = 0.10, t = 2.63, *p* < 0.01) on total attachment to peers (F = 29.49, *p* < 0.001, adjusted R^2^ = 0.08), indicating that adolescents that were female, and athletes, were more attached to peers.

### 3.2. Qualitative Phase Adolescents’ Responses

In the qualitative phenomenology follow up, the analysis resulted in the emergence of three major themes: (1) adolescents’ attachment to sport, (2) adolescent attachment to parents, and (3) adolescents’ thoughts about parents (Table 4).

#### 3.2.1. Adolescents’ Attachment to Sport

This major theme contained several themes. The first was the expression of adolescent emotions in sport.

##### The Expression of Adolescent Emotions in Sport

The adolescents talked about the importance of sport, its contribution to their lives, the reasons that motivate them to play, and feelings experienced about sports. Children enjoy participating in sport: ‘Eventually this gold or silver there is not real, or these cups, but still it means very much because you put a lot of effort into them.’ (G1) Another participant indicated: ‘It is a certain area where you feel good enough in, that is because nobody wants to do what he cannot do; everybody, I think, wants to be somewhere where they can achieve and feel somewhat superior to someone else.’ (T3)

##### Attachment to the Coach

The adolescents emphatically voiced how much the coach means to them. The adolescents identify coaches ‘as parents’. Indeed, sometimes coaches become more important than parents for them: ‘I told her (mother), that you can say whatever you want, but I will listen to the coach because the coach is like a father to me, and for me, he is now much more important than you’. Another participant indicated that it is very important for coaches to be not just technically proficient, but also have the skills and education to provide a holistic training program to help children develop as players and people. For example, one athlete explained: ‘You spend a lot of time with them. They teach, educate you, just like your parents. I think that coaches are your second parents.’ (L4) Overall, in the adolescent period, a period in which the teenagers are performing sport associate their life goals with sport goals, the coach seems to be the right person who knows how to achieve that goal and is a professional helper on that road. In this period, the coach is just like their life coach: ‘during all these 10 years I can say that he raised me up.’ (G1)

##### Price of Youth Sporting Life—Lost Friends

The third theme is the price paid for being an athlete. The most painful aspect for adolescents is losing friends. The participation of parents in their sport is not so significant to adolescents. Sport, coaches, and friends are more important to them: ‘For me, friends are very important; I try very much to give this time to them. And especially in summer, I did not want to leave them.’ (G1) Communication between close friends decreases and loss of a common view occurs: ‘And well, it’s a pity. When you come to school, they ask how your weekend was? Well competition again, this and that … you know, nobody seems interested.’ (C2) As another adolescent athlete discussed: ‘Everybody used to do something, and I was in the gym, so you do not have anything in common with them.’ (T3)

#### 3.2.2. Adolescent–Parent Attachment

Our research revealed that parental involvement in youth sport could bring together parents and children, but it is not something that is necessarily desirable for children. The adolescents tolerated parents’ involvement as long as they were younger because the parents helped them enter the sport, and it was the parents’ first role in children’s sporting activities.

##### The First Role of Parents in Children’s Sport

Discussing how they started sports activity, the adolescents remember being taken to the first training classes by their parents when they were young. Today, they understand that when they were younger, they would not be able to participate in sports without their parents’ involvement: ‘When I was about 5 years old, I was recognized to be hyperactive, I could not stay in one place, so my parents decided to take me to judo.’ (S15) friends to their athletic child. Athletes expressed a deep emotional bond to their parents with gratefulness to them.

##### Trust

‘Trust is the biggest driving force in the parent–athlete relationship.’ (C2) ‘If I don’t trust my parents, I can not rely on them, there is no relationship and communication. There is no respect. Relationship is over.’ (C2) Adolescents athletes highlighted that the feeling of trust is the most important in the parent–athlete relationship in sport. There has to appear to be psychological parental support, recognition of the child’s personal needs in sport, to let the child decide and make their own decisions.

##### Communication

Adolescents engaged in sport admit that then they participate in sport, communication in the family also changes. The common themes, such as communication about competitions, pre-competition mood, post-competition attitude, training session themes, victories and loses, themes appear. Some teenagers say that the new activity ‘brought us closer.’ (F13) ‘Basically, perhaps there was no competition that we would come back from and keep silent, we always hurried to tell them everything, and they always listened attentively, everybody would communicate.’ (L4) However, adolescents expressed the need of mutual understanding in a youth sport environment: ‘If a parent wants a child to achieve in sport and to do better in sports, but the child does not, — it can’t be this way. It is actually my decision. The mutual relationship, and communication would help.’ (S15)

##### Alienation

‘Maybe too much interfering. Such exaggerated attention that you’re trying to relax, you have half an hour break, sometimes, dad is like waving, trying to talk.’ (C2) Another athlete indicated: ‘Dad, I remember him sitting in the gym and just watching me working; it is clear that it was shocking for me because he would shout at me and sometimes just empathize as he would be in the role of the coach.’ (G1) These adolescent athletes’ thoughts are based on long-term experience in sport. Adolescents athletes explained that sport has not brought them closer but separated them from their parents. They noted they do not need the support of their parents and that such support only hinders them. One adolescent said, ‘Well, during the competitions, it makes no difference at all whether they support me or not.’ (T3) However, the financial support of parents was critical: ‘Well, I’ll tell you bluntly in fact, if it were not for my parents, there would be no money to go to these competitions. That is the truth. Maybe I am rude, but that is the truth.’ (G1)

#### 3.2.3. Adolescents’ Thoughts about Parents

A third major theme came out of some interesting examples of adolescent athletes’ ideas about what level of parental involvement would be acceptable to them. In this part of the interview with adolescent athletes, their vision and desires regarding parental support in sport were revealed: ‘While the child is small, I believe you have to show him everything. You shouldn’t say that he will attend basketball and that’s final, or football for example. Maybe he was born to play the piano.’ (D5) Based on adolescent athletes’ thoughts, parents should ask fewer questions and offer supportive comments: ‘Parents should not put pressure but trust [smiles], not to overdo not to ask too much if there is no wish to speak.’ (C2) ‘To ask less about the child’s psychological state, just ask easy questions.’ (C2) Parents have to trust their child and allow him or her to decide for him or herself: ‘There should be a consensus. The child must be allowed to make personal decisions. Parents need to be involved but not too much.’ (L4)

## 4. Discussion

The purpose of this study was to examine the relationship between participation in youth sport and adolescent–parent attachment. Three factors were identified in the quantitative stage of the study namely, trust, communication, and alienation, which allow prediction of the attachment of adolescent athletes to their parents. Furthermore, a qualitative analysis was conducted to better understand the experience of athletes, to look at the study results in all aspects, and to paint a complete picture of the research. The overall results of the quantitative and qualitative stages were integrated in the scheme of the outcomes of the entire study (see Figure 1).

### 4.1. Trust

It should be noted that in the quantitative stage of the study, adolescent athletes’ trust in parents was the most important (they found alienation from parents less important, and at least as important communication with parents). The trust in parents expressed by adolescent athletes can be important to their sporting activities, where relations between children and parents, mutual understanding, and friendship are formed, merging into the concept of a parent–child relationship. In the first major theme arising from conversations with adolescents, ‘adolescents’ attachment to sport’ and in its first theme, ‘expression of emotions in sport’, a general tendency in their opinions could be observed: they talked very fondly about the sport and how much it means to them. The sport has become a part of their life. The adolescents involved in the study show trust and attachment to sport, and their love for the particular sport of choice. Adolescents are grateful to their parents for encouraging them to enter the sport, not only in the spoken thoughts, but also in the unintentionally expressed non-verbal emotions during the conversation. In the second major theme, ‘adolescent–parent attachment’, which along with trust explains recognition of the child’s personal needs, psychological support, parent interest in youth sport, positive involvement, and support to survive in a competitive context, was described by athletes as trust in their interpersonal relationships. In their ethnographic study, Stefansen et al. (2016) [9] revealed that parents see involvement in sport as a way to connect with the child emotionally and to further the child’s development. They interpreted the significance of sport in the parent–child relationship as related both to the normalization of youth sport that the parents experienced when they grew up, and to the new cultural ideas of parenthood that they encounter as adults. Researchers found that there are tensions embedded in this new form of parenthood that is particularly evident in what they call ‘deep involvement’. The third meta-theme, ‘adolescents’ thoughts about parents’, reveals adolescent athletes’ visions and thoughts about what kind of parental involvement would be acceptable to them. In their words, there is an evident desire for their parents to have more confidence and trust to allow them to choose and decide [10]. Study shows that the experience of friendships from youth sports is influenced by effectively used models of cognition and emotions, which they ‘bring with them’ from the basis of previous relationships with parents. The study results by [18] showed that early adolescents are more attached to their parents. Also, adolescent involvement in competitive sport had no effect on their peer attachments, which indicates that a close relationship with other sport participants does not predict greater peer attachment. Our study shows that adolescent athletes who enter middle adolescence show a strong decrease in parental attachment and become more attached to their peers.

### 4.2. Communication

On this scale, adolescent athletes’ expression of communication with parents appears to be the least important. Also, qualitative results explain these results and demonstrate that adolescent athletes avoid active communication with their parents; however, in certain moments, communication between parents and adolescent athletes is extremely important. The findings of the phenomenological analysis help to explain these results. In the study of the experience of adolescent participation in sporting activities and their relationships with their parents, the adolescent athletes’ attitude to communication with parents in sporting activities is conveyed in the major theme ‘parent–adolescent attachment’. Looking back to early childhood, some participants relate the start of this kind of communication to their entry into the sport. They speak warmly about their parents’ long-term efforts to support them. Children appreciate being able to communicate easily with parents. They become closer to their parents while talking about sporting issues and disappointment about failures, as well as the joys of sporting achievements. Athletes highlighted that mutual understanding, respect of athlete’s opinion, and particularly the heard voice of the child is important in maintaining positive relationships between the child and the parent. In the context of communication between parents and children in sport, it should be noted that the qualitative research highlighted the encouraging role of coaches in adolescent–parent communication in sporting environments. All children who participated in the study noted that with the efforts of the coach, communication between children and parents in sport became closer. Studies by [2,12] have also emphasized the importance of cooperation between parents and coaches for children in sport.

### 4.3. Alienation

Despite the fact that in the quantitative stage of study adolescent athletes indicated stronger attachment to parents and peers, trust in them, and closer communication compared to adolescents not involved in sport, their responses showed statistically significant differences in the alienation scale. The results of the qualitative study complement and explain this phenomenon. Qualitative results explain how and why adolescent–parent trust, communication, and alienation appears and what are the reasons for the parent–athlete interpersonal relationship phenomenon. The interviews with adolescent athletes in the qualitative study revealed that the attachment of adolescent athletes to parents decreases with increasing sporting experience. Adolescents tend to rebel, try to escape from the influence of their parents, demonstrate individuality, and sport becomes another premise of estrangement from their parents. As the experience in sport increases, they become more attached to it, as well as their coach and the competition. They dive wholeheartedly into sports activities, where they can achieve their personal goals and express themselves in the way that only they understand and not their parents. Sport is conceived as a personal space. It could be explained, based on [30], a mixed-methods study, which revealed that the meaning youth sport participants gave to their sport involvement (i.e., goals, values, and purposes) and the features of the social-environment they perceived to be important differed between the four subtypes of perfectionism. Researchers concluded that youth sport participants demonstrate different subtypes of perfectionism and vary in their experiences of youth sport. Problems regarding adolescent–parent alienation in youth sporting activities were revealed in the interviews with adolescents under the theme ‘parent–adolescent attachment’. Young athletes, in turn, identified parental emotions expressed during competitions as barriers to establishing closeness to their parents. All adolescents mentioned that tactless behavior by parents is not encouraging, disturbs concentration, and makes it hard to get in the appropriate pre-competition mood. Research by [8] supports our findings and has illuminated debriefing in the post-game setting as another conduit through which parents can either positively or negatively contribute to the youth sport experience. The complex phenomenon of parenting in youth sport highlights the fact that it is possible to build a positive interpersonal relationship through close relationships and secure attachment in the family. In relation to this, the hypothesis of our study that athletes are more attached to their parents compared with non-athletes was confirmed, and based on the current study results, future research could focus on a longitudinal adolescent–parent interpersonal relationship investigation. Furthermore, this study evaluated adolescent attachment to parents measured by two parents, rather than by distinguishing between mothers and fathers. From the practical perspective, the results and conclusions of this study may help managers of sport clubs, organizations, school staff, and educators to better understand how parents—by creating positive and supporting relations in sporting activities of their teenage children—may add to the achievement of the goals and objectives, and in this way educate democratically-minded youth ready to actively engage in social and public life. This study is relevant and has a practical value for the promotion of the positive development of adolescents and responsible parenting. It opens a new view for professionals (coaches, sport educators, sport psychologists) working with athletes and their parents to the variety of experiences and through the knowledge gained to increase the wellness of athletes and their parents. Thus, the clearer aspects of family attachment and experiences of athlete adolescents in their relations with parents may help to improve the skills not only of athlete adolescents but also of their parents and coaches.

### 4.4. The Integration of the Mixed-Methods Study

Understanding parent–adolescent interpersonal relationships in youth sport requires a detailed explanation; therefore, the mixed methods study was designed to reveal the study aims. As the mixed-methods study requires a detailed visual joint display, the integration scheme is presented in Figure 1. The scheme illustrates a visual display of the integration of mixed survey results. The diagram illustrates how three factors of quantitative research: trust, communication and alienation are complemented by qualitative data.

Overall, the quantitative study results showed that adolescents scored the highest on trust, and the qualitative study explains such results. A major theme ‘adolescent attachment to sport’ with themes ‘expression of adolescent emotions in sports’; and ‘attachment to coach’ highlights trust in a chosen field, especially trust and attachment to their coach. Adolescent athletes see their coach as a second parent, and those trust emotions to a coach become even stronger than to parents. The second major theme ‘adolescent–parent attachment’ and the theme ‘the first role of parents’ also highlight adolescent feelimgs of trust in their parents. The first entry into a sport with the help of parents created a strong feeling of trust that adolescents are being directed in the right direction. However, as adolescents become older, their trust in parents lessens.

Moreover, the quantitative results showed that adolescents scored less on alienation and least on communication scales. The alienation appears in such themes as ‘adolescent attachment to sport’ with a theme ‘price of youth sporting life: lost friends’ and explains quantitative results, as there were statistically significant differences on alienation peer and parent scale. This theme explains how much friends mean to athletes, and the strict sport environment is one factor that separates adolescents from school friends. Adolescents also mentioned that peers in a sport environment are different, they seek the highest results and they are more competitors than real friends. In addition, the alienation from parents appears in a second major qualitative theme ‘adolescent–parent attachment’ with themes ‘communication’ and ‘alienation’. The alienation in communication theme appears when adolescents talk about a decreasing need for mutual communication. Parents disturb them too much, or interfere. The alienation theme explains such topics as overinvolved parents and pressure perceived by parents which is not welcomed by adolescent athletes.

The adolescents scored least on the communication scale. The qualitative themes explain and show that communication is important to athletes with a coach, they expressed deep feelings and very sensitive moments during the interviews. Adolescent athletes explained that communication could be reached through a strong feeling of trust.

## 5. Conclusions

The analysis of the adolescent–parent interpersonal relationship revealed that athlete adolescents’ relations and attachment to parents are more intensively expressed in all scales: trust, communication and alienation, compared to non-athlete adolescents. Interviews with adolescent athletes revealed that parent–adolescent interpersonal relationships and attachment to parents are more important in the early period of sporting life, and become less appreciable or unwelcome when children gain sporting experience. The present study indicated that the form and degree of parental involvement in children’s sporting activities impact the effectiveness of parent–athlete interpersonal relationships. The degree and form of parental involvement in children’s sport chosen by the parents are not always appropriate and encouraging, and they are not always supportive of adolescents’ opinions. Future research could investigate adolescent–parent attachment by mothers and fathers separately. Parental involvement in youth sport intervention programs could be developed based on this study’s results and this would add scientific value to the background of parenting in youth sport.

## Figures and Tables

**Table 1:** Demographic characteristics of adolescent athletes participating in qualitative interviews.

Participants	Gender		Race	Age	Sport type	Sport Experience (years)
15	Eight female	Seven male	White	16	Individual	8.8

**Table 2:** Correlations and descriptive statistics.

Variables	Mean (M)	Standard Deviation (SD)	1	2	3	4	5	6	7	8	9	10
1.Trust in parents	23.38	3.38	**0.82**									
2. Communication with parents	15.91	3.01	0.71 **	**0.76**								
3. Alienation from parents	16.36	2.58	0.63 **	0.50 **	**0.74**							
4. Total parent attachment	22.94	4.78	0.81 **	0.86 **	0.22 **	**00.91**						
5. Trust in peers	25.63	3.79	0.32 **	0.31 **	0.27 **	0.27 **	**00.83**					
6. Communication with peers	18.24	3.60	0.22 **	0.30 **	0.14 **	0.27 **	0.76 **	**0.82**				
7. Alienation from peers	9.57	1.45	0.19 **	0.16 **	0.33 **	0.06	0.54 **	0.39 **	**0.69**			
8. Total peer attachment	34.29	6.32	0.27 **	0.32 **	0.17 **	0.30 **	0.91 **	0.93 **	0.32 **	**0.89**		
9. Involvement in sport	-	-	0.09 *	0.07	0.06	0.07	0.09 *	0.05	0.10 *	0.06		
10. Sport experience	4.45	2.48	− 0.04	−0.04	−0.03	0.07	−0.03	−0.06	0.02	−0.05	0.14 *	
11. Gender	-	-	0.10 *	−0.01	−0.12 **	0.05	−0.18 **	−0.33 **	−0.11 **	−0.27 **	−0.13 **	0.03

Note. * *p* < 0.05; ** *p* < 0.01. Scale reliabilities (α) are in bold type on the diagonal.

**Table 3:** Differences between adolescent participation in sports activities and their attachment to parents and peers.

**Participation in Sport**							
Variables	Athletes (n = 209)		Non-athletes (n = 439)		t	p	Cohen d
	M	SD	M	SD			
Trust in parents	23.76	3.22	23.20	3.43	2.01	0.05	0.18
Communication with parents	16.22	2.87	15.76	3.06	1.79	0.07	0.16
Alienation from parents	16.59	2.62	16.24	2.56	1.59	ns	
Total parent attachment	23.39	4.47	22.72	4.90	1.66	ns	
Trust in peers	26.13	3.57	25.39	3.86	2.33	0.02	0.20
Communication with peers	18.51	3.28	18.10	3.74	1.35	ns	
Alienation from peers	9.77	1.37	9.47	1.48	2.45	0.02	0.21
Total peer attachment	34.87	5.92	34.01	6.49	1.61	ns	

**Table 4:** Experience of adolescent participation in sporting activities and relationship with parents: theme table.

Major Themes	Themes	Sub-Themes
**Adolescent attachment to sport**	Expression of adolescent athletes’ emotions	Enjoyment of sport Emotions caused by competitions Emotions caused by injuries Expression of victory and defeat
	Attachment to coach	Coaches as second parents Coach as a friend A profile of an ideal coach
	Price of youth sporting life: lost friends	Lack of time for peers Alienating peers Nothing in common with peers
**Adolescent–parent attachment**	The first role of parents	Supporter Moderator Friend
	Trust	Recognition of the child’s personal needs Psychological support Parent interest in youth sport Positive involvement Believe in the child Support to survive in competitive context
	Communication	Mutual understanding Respects athlete’s opinion Positive verbal communication Positive non-verbal communication Respect Decreasing need for communication
	Alienation	Pressure Over-involved parenting Poor behaviour at competitions Parents‘ critique Interferes too much
**Adolescents’ thoughts about parents**	Adolescents’ vision	Let the child decide for him- or herself Do not put pressure Trust the child Support Communicate Motivate Be positively involved

## References

[1] Dorsch T.E., Smith A.L., McDonough M.H. (2009). Parents’ perceptions of child-to-parent socialization in organized youth sport. J. Sport Exerc. Psychol..

[2] Holt N.L., Knight C.J. (2014). Parenting in Youth Sport: From Research to Practice.

[3] Knight C.J., Holt N.L. (2014). Parenting in youth sport: Understanding and enhancing children’s experiences. Psychol. Sport Exerc..

[4] Nunomura M., Oliveira M.S. (2013). Parents’ support in the sports career of young gymnasts. Sci. Gymnast. J..

[5] Sapieja K.M., Dunn J.G., Holt N.L. (2011). Perfectionism and perceptions of parenting styles in male youth soccer. J. Sport Exerc. Psychol..

[6] Sanchez-Miguel P.A., Leo F.M., Sanchez-Oliva D., Amado D., Garcia-Calvo T. (2013). The importance of parents’ behavior in their children’s enjoyment and motivation in sports. J. Hum. Kinet..

[7] Kaplanidou K., Gibson H.J. (2012). Event image and traveling parents’ intentions to attend youth sport events: A test of the reasoned action model. Eur. Sport Manag. Q..

[8] Elliott S.K., Drummond M.J.N. (2017). Parents in youth sport: What happens after the game?. Sport Educ. Soc..

[9] Stefansen K., Smette I., Strandbu A. (2016). Understanding the increase in parents’ involvement in organized youth sports. Sport Educ. Soc..

[10] Carr S. (2014). Attachment in Sport, Exercise, and Wellness.

[11] Bailey R., Cope E.J., Pearce G. (2013). Why do children take part in, and remain involved in the sport? A literature review and discussion of implications for sports coaches. Int. J. Coach. Sci..

[12] Wolfenden L.E., Holt N.L. (2005). Talent development in elite junior tennis: Perceptions of players, parents, and coaches. J. Appl. Sports Psychol..

[13] Reeves C., Nicolls A., McKenna J. (2009). Stressors and coping strategies among early and middle adolescent Premiere League academy soccer players: Differences according to age. J. Appl. Sport Psychol..

[14] Lisinskiene A. (2016). Educational Interaction between Adolescents and Parents in Sporting Activities. A Dissertation.

[15] Belsky J., Fearon R.M.P. (2002). Infant-mother attachment security, contextual risk, and early development: A moderational analysis. Dev. Psychopathol..

[16] Bowlby J. (1969). Attachment and Loss.

[17] Bowlby J., Cohen R.S., Weissman S.H., Cohler B.J. (1982). Caring for children: some influences on its development. Parenthood.

[18] Sukys S., Lisinskiene A., Tilindiene I. (2015). Relation between adolescents’ participation in sports activities and their attachment to parents and peers. Soc. Behav. Personal..

[19] Nawaz S., Gilani N. (2011). Relationship of parental and peer attachment bonds with career decision—Making self-efficacy among adolescents and post-adolescents. J. Behav. Sci..

[20] Horn T.S., Horn J.L., Tenenbaum G., Eklund R.C. (2007). Family influences on children‘s sport and physical activity participation, behavior, and psychosocial responses. Handbook of Sports Psychology.

[21] Creswell J.W., Clark V.L.P. (2011). Designing and Conducting Mixed Methods Research.

[22] Creswell J.W., Plano Clark V.L., Guttman M., Hanson W., Tashakkori A., Teddlie C. (2003). Advanced mixed methods research designs. Handbook of Mixed Methods in Social and Behavioral Research.

[23] Creswell J.W. (2013). Qualitative Inquiry and Research Design: Choosing among Five Approaches.

[24] Smith J.A., Flowers P., Larkin M. (2009). Interpretative Phenomenological Analysis: Theory, Method and Research.

[25] Gullone E., Robinson K. (2005). The inventory of parent and peer attachment-revised (IPPA-R) for children: A psychometric investigation. Clin. Psychol. Psychother..

[26] Armsden G.C., Greenberg M.T. (1987). The inventory of parent and peer attachment: Individual differences and their relationship to psychological well-being in adolescence. J. Youth Adolesc..

[27] Sukys S. (2004). Interrelation among behavioral decisions of adolescents, their gender, and physical activity. Educ. Phys. Train. Sport.

[28] Sacks D., Westwood M. (2003). An approach to interviewing adolescents. Paediatr. Child Health.

[29] Yardley L. (2000). Dilemmas in qualitative health research. Psychol. Health.

[30] Mallinson-Howard S.H., Knight C.J., Hill A.P., Hall H.K. (2018). The 2 × 2 model of perfectionism and youth sport participation: A mixed-methods approach. Psychol. Sport Exerc..

